# Utilization of Fish Byproduct in Edible Films: A Sustainable Approach to Improving Fillet Shelf Life

**DOI:** 10.1002/fsn3.70731

**Published:** 2025-08-04

**Authors:** Gonca Alak, Ayşe Kara, Rahime Altıntaş, Melda Şişecioğlu

**Affiliations:** ^1^ Department of Seafood Processing Technology, Faculty of Fisheries Ataturk University Erzurum Türkiye; ^2^ Department of Seafood Processing, Faculty of Fisheries Recep Tayyip Erdoğan University Rize Türkiye; ^3^ Department of Molecular Biology and Genetics, Faculty of Science Ataturk University Erzurum Türkiye

**Keywords:** coating, fillet quality, lectin, preservative, shelf life

## Abstract

Seafood has attracted attention in recent years. In this context, it is aimed to investigate the effectiveness of lectin obtained from mucus containing many defense molecules, including immunoglobulins, on fillet quality as an enriching component in biofilm coatings, and to evaluate the effectiveness of aquaculture waste as a preservative in the food industry within the scope of green chemistry applications, sustainability, and green product targets. In line with this objective, lectin (LCT) is purified from mucus by three‐phase partitioning (TPP) method in the first phase; then, in the second phase, chitosan (Ch) based coating solution is enriched with LCT (0.04 mg/L); and in the last phase, fillets are coated with this solution and fillet quality parameters are monitored by microbiological, chemical, and sensory analyses at certain intervals during 12 days of storage (at +4°C ± 1°C). According to the findings, the coating enriched with LCT caused relative changes in appearance, color, and a* value among sensory tests, but these changes are not statistically significant. Based on the findings of this modeling study, the LCT‐enriched coating has significant potential as an active coating for the preservation of food quality. This indicates its suitability for application as a green active packaging solution.

## Graphical abstract

LCT has been used as an active coating in the protection of food quality. The biological activities of LCT purified from mucus are a preservative for the seafood processing. LCT purified from mucus for the aquaculture processing sector can innovatively be used.

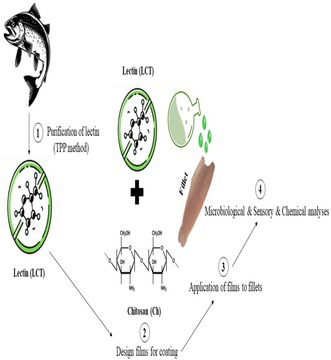

## 1 Introduction

There are many different and complex defense mechanisms fish have in order to protect themselves from different stressors such as bacterial or viral infections. Fish skin mucus is essential as it acts as the first line of physical or chemical defense against pathogens. Although the composition of mucus varies among fish species, it has a wide range of functions in all fish including disease resistance, protection, respiration, ionic and osmotic regulation, reproduction, excretion, communication, feeding, and nesting. Fish skin mucus has two functions as an important component of the innate immune system. First, it is continuous production and regular secretion prevent the adhesion of pathogens, the stable colonization of potentially infectious microbes, and the invasion of parasites. Second, it includes immune parameters such as lectins, pentraxins, lysozyme, complement proteins, antibacterial peptides, and IgM (Dik et al. 2024; Zhao et al. 2024).

Chromatographic methods are extensively used for the purification of lectin from mucus (Sun et al. 2019). Besides these methods, as an alternative to traditional methods, the three‐phase separation (TPP) method, which was first developed by Lovrien et al. for the isolation of enzymes and proteins from multicomponent systems, is a new one‐step, low‐cost, and effective bioseparation technique (Gagaoua and Hafid 2016). TPP, which exhibits a complex structure influenced by various factors, such as osmotic stress, ionic strength, cosmotropy, aggregation interactions, and interfacial tension, is a physicochemistry‐based technique. In the TPP system, proteins show different behaviors depending on their pI, source, molecular weight, and temperature (Dennison and Lovrien 1997; Saxena and Baker 2010). Although initially developed as an “upstream” technique, it is often used as a “downstream” method for isolating substances at the milliliter volume scale (Ketnawa et al. 2017). TPP is a simple technique in which a salt (usually ammonium sulfate) and an organic solvent (usually t‐butanol) are added to an aqueous protein solution. Three phases consisting of a protein‐enriched interfacial precipitate between the lower aqueous and upper organic phases are formed in less than an hour (Roy and Gupta 2002; Boucherba et al. 2017).

Lectins (LCTs), glycoproteins, or proteins of non‐immune origin can agglutinate cells or precipitate glycoconjugates via carbohydrate‐specific binding sites. Previous studies have revealed that lectin has many functions such as protection, biological nitrogen fixation, mitogenesis, and immune modulation (Sun et al. 2019). Some LCTs, natural proteins, exhibit strong antimicrobial effects by binding to carbohydrates on microbial surfaces. The oligomerization state of lectins can affect their biological activities and maximum binding capacities; the interaction between lectin polypeptide chains can alter the carbohydrate‐lectin binding dissociation rate constants. The antimicrobial mechanisms of lectins include their ability to form pores, followed by cell permeability changes and interactions with bacterial cell wall components (Breitenbach Barroso Coelho et al. 2018).

Edible film and coating technology has emerged with the increasing demands for quality food consumption, the search for new technologies for food preservation, wastes from artificial polymers, and the idea of producing new technologies from sustainable resources. Chitosan is derived from chitin; it is an edible polymer isolated from the shells of shellfish. It is a non‐toxic and environmentally friendly natural product. Chitosan, a high molecular weight cationic polysaccharide produced by the deacetylation of chitin, is widely applied in post‐harvest processes due to its excellent film‐forming, antifungal, antibacterial, and biochemical properties. Recently, chitosan has gained significant interest due to its biological activities, including antimicrobial (Tsai et al. 2004), antitumor (Tokoro et al. 1988), antioxidant (López‐Caballero et al. 2005), and hypocholesterolemic functions (Sugano et al. 1980) and it possesses bacteriostatic and bactericidal properties. Therefore, chitosan is highly recommended as a polymer for producing edible film coatings (Chien et al. 2007; Sree et al. 2020).

In this regard, aquaculture, one of the most dynamic sectors in the global food system, is surprisingly underrepresented in abundant literature on food policy. Aquaculture is one of the fastest growing sectors in the world, and it has significant potential in terms of rich‐content waste and health‐related products. The utilization of these industrial fish wastes has attracted the attention of the scientific community due to their integrated benefits such as waste management and resource recycling. These wastes have become a promising raw material due to the presence of more biodegradable organic materials suitable for different industrial uses such as biodiesel, biogas, animal feeds, dietary ingredients, food packaging, and catalysts in fields such as medicine, dentistry, pharmacy, cosmetics, and agriculture. An unmet need exists to utilize these by‐products by converting them into functional and nutritious ingredients. Following this lack, this study aims to provide preliminary data for creating infrastructure solutions for obtaining high value‐added products by utilizing animal wastes and to determine the potential for practical use.

As a result of the rapid increase in the world population, the creation of new food resources and the utilization of existing resources, as well as conscious consumer trends in fish preservation, the development of additives that preserve the freshness of fish and do not deteriorate the chemical structure of fish, and especially the studies on packaging have accelerated (Alak et al. 2023; Ortizo et al. 2023; Vazquez‐Ayala et al. 2024). This process has put tremendous pressure on the agriculture and food sector. Although the sustainable development goals of the United Nations propose to take strict measures to create a balance in these sectors through research and government policies (Ortizo et al. 2023; Vazquez‐Ayala et al. 2024), the Sixth Environmental Action Plan has made the prevention of waste at source as well as the promotion of recycling, the use of unavoidable waste as a resource and the extraction of additional natural resources the main elements of the European Union waste management policies (Korkmaz 2021).

This study aims to develop a new generation of products through green chemistry applications to address growing concerns about food preservation. The focus is on extending the shelf life of fish fillets by utilizing the antimicrobial properties of lectins during the preservation process without relying on synthetic additives that may alter the chemical structure of the fish. Specifically, the research aims to purify and characterize lectins extracted from rainbow trout mucus and evaluate their potential impact on the physical, chemical, microbiological, and sensory quality parameters of fillets. These lectins will be incorporated as additives in biofilm coating solutions and tested under in vitro conditions.

Considering the aforementioned process and current consumer trends, this study aims to serve as a model by contributing to the literature in several key areas. These include exploring the potential of aquaculture wastes as value‐added products, assessing their impact on the shelf life and quality of foods, and introducing them into the food industry as natural preservatives. Additionally, the study seeks to advance the development of edible films and evaluate their effects on food quality.

## 2 Materials and Methods

### 2.1 Purification of Lectin From Mucus With TPP

Mucus was obtained from broodstock rainbow trout ( *Oncorhynchus mykiss* ) in Atatürk University Faculty of Aquaculture, Inland Fish Application and Research Centre. In this study, 10 fish were used and placed in a ladle and encouraged to secrete mucus, and approximately 4–5 mL of mucus was taken from each fish, thus 50 mL of mucus was collected and centrifuged (6000 rpm for 5 min) to remove impurities. A three‐phase formation was observed on the top as the organic phase, in the middle as precipitate, and at the bottom as the aqueous phase. The supernatant and subphases were carefully removed using a micropipette. The middle phase, likely to contain precipitated lectin, was collected and dissolved in pH 7.4 sodium phosphate buffer to determine protein content and haemagglutination activity.

Ammonium sulfate (20%–50%–80% w/v) was initially added to the supernatant (2 mL). Subsequently, t‐butanol was gradually added in different ratios (1.0:0.5, 1.0:1.0, 1.0:2.0). The aqueous/organic system was vortexed and incubated at room temperature for 1 h. A three‐phase formation was observed on the top as the organic phase, in the middle as precipitate, and at the bottom as the aqueous phase. The supernatant and subphases were carefully removed using a micropipette. The middle phase, likely to contain precipitated lectin, was collected and dissolved in pH 7.4 sodium phosphate buffer to determine protein content and haemagglutination activity.

Afterward, the mixture was subjected to dialysis (MWCO: 3000 Da) against sodium phosphate buffer pH 7.4 for 48 h at +4°C. Then it was concentrated to 1000 mL, and the partially purified lectin was lyophilized.

### 2.2 Optimization of TPP Conditions by Response Surface Method (RSM)

Optimization was performed by applying the Box–Behnken experimental design method (BBD). By this target, the ammonium sulfate salt was added to the medium, and the amount of t‐butanol organic solvent and pH variables were firstly optimized to obtain the protein amount with the highest yield. The response surface design of three different factors at −1, 0, and +1 levels was created with the BBD, and the levels and coded values of each factor investigated are given in Table 1. Salt ratio, homogenate: t‐butanol ratio, and pH, important TPP parameters for the purification of lectin protein, are independent variables. A total of 15 experiments were created in the design, and the center point is repeated several times to test its repeatability. All experiments were carried out in three repetitions. Minitab 18 trial version was used in experimental design, determination of coefficients, data analysis, and drawing of graphs. The equation of the model is as follows:

$$y=\left(454.8+66.6A-26.5B+49.0C-139.9A*A+89.7B*B-96.8C*C+94.7A*B-35.9A*C-11.5B*C\right)$$

**TABLE 1:** Independent variables and levels of lectin protein.

Independent variables	Unit	Low level	Mean	High level
		−1	0	1
A: Ammonium sulfate	%	20	50	80
B: t‐butanol	—	0.5	1	2
C: pH	—	6	7	8

### 2.3 Haemagglutination Activity and Protein Determinations

It was carried out with 25 μL of 5% rabbit blood and 25 μL of mucus extract at different concentrations (1/1–1/512) (10 mM phosphate buffer [pH: 7.4] containing 150 mM in the dilution process). The haemagglutination activity is determined by incubating 96% microplates at room temperature for 1 h. In this phase, the lowest concentration at which collapse occurred is taken as a basis. The lowest inhibition concentration is then determined using the tube with the lowest concentration (Okamoto et al. 2005). The Bradford method uses BSA as a protein standard to determine protein concentration in the samples (Bradford 1976).

The yield (*Y*) was defined as the ratio of hemagglutinating activity in the interfacial phase (HA_I_) to that in the homogenate (initial lectin solution) (HA_H_), expressed as a percentage as follows:

$$Y=\frac{{\mathrm{HA}}_{I}.V_{I}}{{\mathrm{HA}}_{H}.V_{i}}\times 100$$

where *V*_I_ and *V*_i_ represent the volumes of the interphase and homogenate, respectively.

Purification factor (PF) was calculated as the ratio of specific activity in the interphase phase (HA_i_/*C*_i_) to homogenate specific activity (HA_H_/*C*_H_) as follows:

$$\mathrm{PF}=\frac{{\mathrm{HA}}_{i}/C_{i}}{{\mathrm{HA}}_{H}/C_{H}}$$

in which *C*_i_ and *C*_H_ represent the total protein concentrations expressed in mg/mL in the interphase phase and the homogenate, respectively.

### 2.4 Polyacrylamide Gel Electrophoresis

In order to check the purity of the lectin purified by the TPP method and to determine the approximate molecular weight, SDS‐PAGE is conducted using 4% stacking and 15% separating gels, a Bio‐Rad Mini Protean II electrophoresis unit, and standard molecular weight markers (Ecotech Biotechnology). Silver staining of the gel is performed according to the described method (Hames 1986).

### 2.5 Trial Design for Coating Solution and Fillet

The research was carried out in the laboratories of Ataturk University and Recep Tayyip Erdoğan University. 360 rainbow trout ( *O. mykiss* ) fillets (average weight 175 ± 5 g) were prepared in a sterile medium and were distributed depending on chance as 30 pieces in each group. The lectin (LCT) was obtained from mucus with TPP. Chitosan was obtained from a commercial company (low viscous chitosan; Sigma‐Aldrich, Israel). After being dissolved in acetic acid as a 1.5% w/v solution (7.5 g of chitosan in 500 mL of acetic acid solution) overnight at room temperature (Alak et al. 2023, 2010), to obtain a homogeneous mixture, the solutions were stirred again in a magnetic stirrer for 24 h at room temperature. Once the dissolution process was completed, chitosan solutions were enriched with LCT by adding to this solution at 0.04 mg/L (Alak et al. 2019). In a study, it was reported that total dietary lectin intake ranges from 0 to 200 mg per day (Lucius 2020). Considering this range value, 0.02% of this value was used in the study. At the same time, groups that were treated without Ch and/or LCT enrichment were considered control groups (Table 2).

**TABLE 2:** Trial groups for the assessment of curative potential of lectin on fillet quality.

Experimental code	Groups	Treatment		Fillet counts
		LCT (mg/L)	Chitosan (w/v)	
Ch	Only chitosan	—	1.5%	30
Ch + LCT	Chitosan + lectin	0.04	1.5%	30
LCT	Only lectin	0.04	—	30
Control	No treatment	—	—	30

### 2.6 Application of Coating Solutions (Films) to Fillets

By applying the dipping method to the prepared film solutions, the fillets were kept in the solution under sterile conditions, and the fillet dipped into the dipping solutions was fully immersed (for 5 min) in order to coat the whole fillet surface (Alak et al. 2023; Wu 2014; Güler et al. 2022) and allowed to air dry for 5 min at room temperature (Karsli et al. 2021). The coated fillets were stored under cold storage (4°C ± 1°C, and 50% relative humidity) for 12 days. Microbiological, chemical, and sensorial analyses were performed to determine fillet quality and shelf life during this storage period at certain intervals (0, 3, 6, 9, and 12 days).

### 2.7 Cold Storage Analyses

#### 2.7.1 Microbiological analyses

The analyses of fillets coated with biofilm solutions prepared at different concentrations and stored under refrigerated conditions were performed for total aerobic mesophilic bacteria, lactic acid bacteria, psychrotrophic bacteria, yeast‐mold, and Enterobacteriaceae bacteria. For the analyses to be performed under sterile conditions, 25 g of sample was taken and homogenized in Stomacher by adding 250 mL of saline water. Appropriate dilutions were prepared from this homogenate, and analyses were performed for each bacterial species by considering specific media and incubation conditions (Table 3) (Atamanalp et al. 2021; Korkmaz et al. 2019).

**TABLE 3:** Bacteria and incubation references.

Bacteria	Agar	Incubation
Total aerobic mesophilic bacteria	Plate Count Agar (PCA, Merck)	37°C for 48 h aerobic
Psychrotrophic bacteria	Plate Count Agar (PCA, Merck)	10°C for 7 days aerobic
Lactic acid bacteria	De Man Ragose Sharpe Agar (MRS, Merck)	30°C for 48 h anaerobic
Enterobacteriaceae	Violet Red Bile Dextrose Agar (VRBD. Merck)	30°C for 48 h anaerobic
*Pseudomonas*	Cetrimide Agar (CFC, Merck)	25°C for 48 h aerobic

#### 2.7.2 Chemical Analyses

The following parameters were considered, as they give quick results and are widely used in monitoring chemical changes occurring in fish muscle during deterioration

- TVB‐N (total volatile basic nitrogen) analysis: After homogenization with TCA solution in Ultra Turrax (IKA WerkTp 18‐10 20,000 rpm) for 5 min, the mixture was filtered with filter paper, following the method described by Malle and Tao (1987). The resulting solution was treated with NaOH and placed in the distillation apparatus. The resulting distillate was titrated until achieving a pink color, and TVB‐N value was calculated as mg/100 g (Atamanalp et al. 2021).
- Determination of TBA (thiobarbituric acid) value: The method given by Tarladgis et al. (1960) was followed, and the samples were homogenized. The 4N HCl solution was added to the resulting homogenate mixture, and the mixture was incubated (35 min at 100°C) and then read at 538 nm wavelength (Shimadzu, UV 160) in a spectrophotometer. The absorbance values obtained were formulated, and the value of TBA was determined as mg MA/kg.
- pH measurement: 10 g of small pieces of samples were taken in parallel, and 100 mL of pure water was added. The mixture was homogenized in Ultra Turrax (for 1 min) and the pH value was measured with a pH meter (Lawless and Heymann 2010; Kara et al. 2025).

#### 2.7.3 Sensory analyses

The analyses were carried out by 10 panelists. The panelists were trained before conducting the sensory evaluation of the fillets, and calibration sessions were held during each sensory evaluation period. The study was reviewed and approved by Atatürk University, and informed consent was obtained from each subject prior to their participation in the study. The panelists evaluated the raw fillets of the treatment groups in terms of color, appearance, and odor. A hedonic scale with a score range of 1–3 (very poor—unacceptable), 4–5 (intermediate), 6–7 (good), and 8–9 (very good) was used for the panelists' evaluations (Kara et al. 2025).

### 2.8 Statistical Analyses

All data were statistically analyzed using GraphPad Prism 8 software. Results are presented as mean ± standard deviation (SD), with statistical significance set at the following levels: ****p < 0.0001, ***p < 0.001, **p < 0.01, *p < 0.05, and ns: non‐significant (*p* > 0.05). Homogeneity tests were applied for all output using generalized linear models. All data were analyzed using two‐way ANOVA followed by Tukey's test. The Shapiro–Wilk method was used to assess the normality of the data.

## 3 Results

### 3.1 TPP and Experimental Design RSM

**FIGURE 1:**
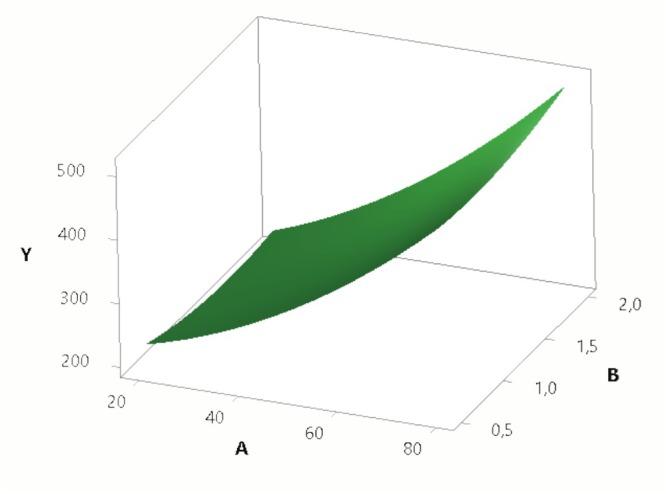
3D graphic images of the combined effects of three variables on mucus lectin protein amount in TPP (A: ammonium sulfate, B: t‐butanol, and Y: protein amount).

As a result of the optimization of the TPP with the RSM, as shown in Figure 1, the *p* value of the linear and quadratic model is found to be significant (*p* < 0.05) in the model selection performed by the response surface method at a 95% confidence interval. Under optimized conditions, parameters determined by the optimization of lectin TPP from fish mucosa were used. The best lectin yield (400%) and 7.3‐fold purification were seen in the interfacial phase in the presence of 80% (w/v) ammonium sulfate saturation with 1.0:0.5 homogenate/t‐butanol ratio (v/v) at pH 7.0 for 2 mL fish mucus homogenate. We obtained approximately 10% lectin in fish mucus.

### 3.2 SDS‐PAGE Output

**FIGURE 2:**
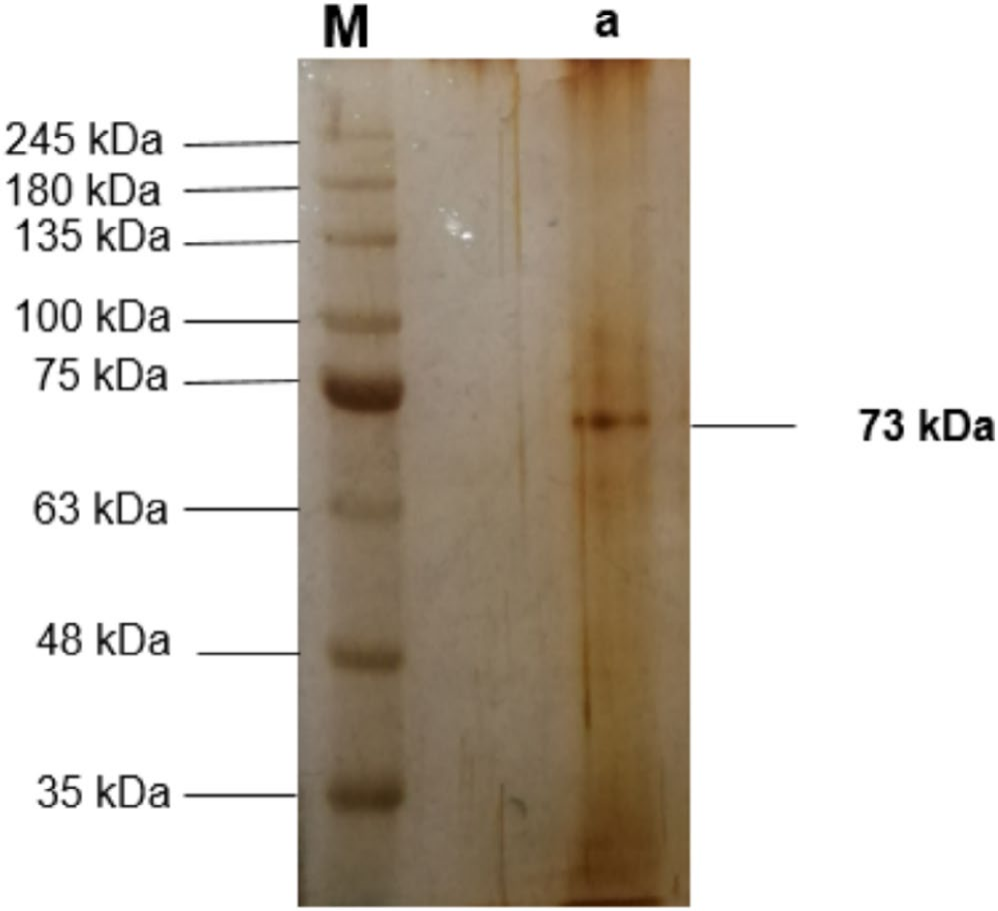
SDS–PAGE of purified mucus lectin. Lane M: Molecular weight marker proteins, Lane M: Marker, and Line a: TPP interfacial precipitate.

The SDS‐PAGE method is used to control the purity and determine the molecular weight of alkaline lectin partially purified from fish mucosa with TPP. The molecular weight of the lectin was determined to be approximately 73 kDa by SDS‐PAGE (Figure 2).

### 3.3 Microbiological, Chemical and Sensory Changes in Fillets Coated With LCT

**FIGURE 3:**
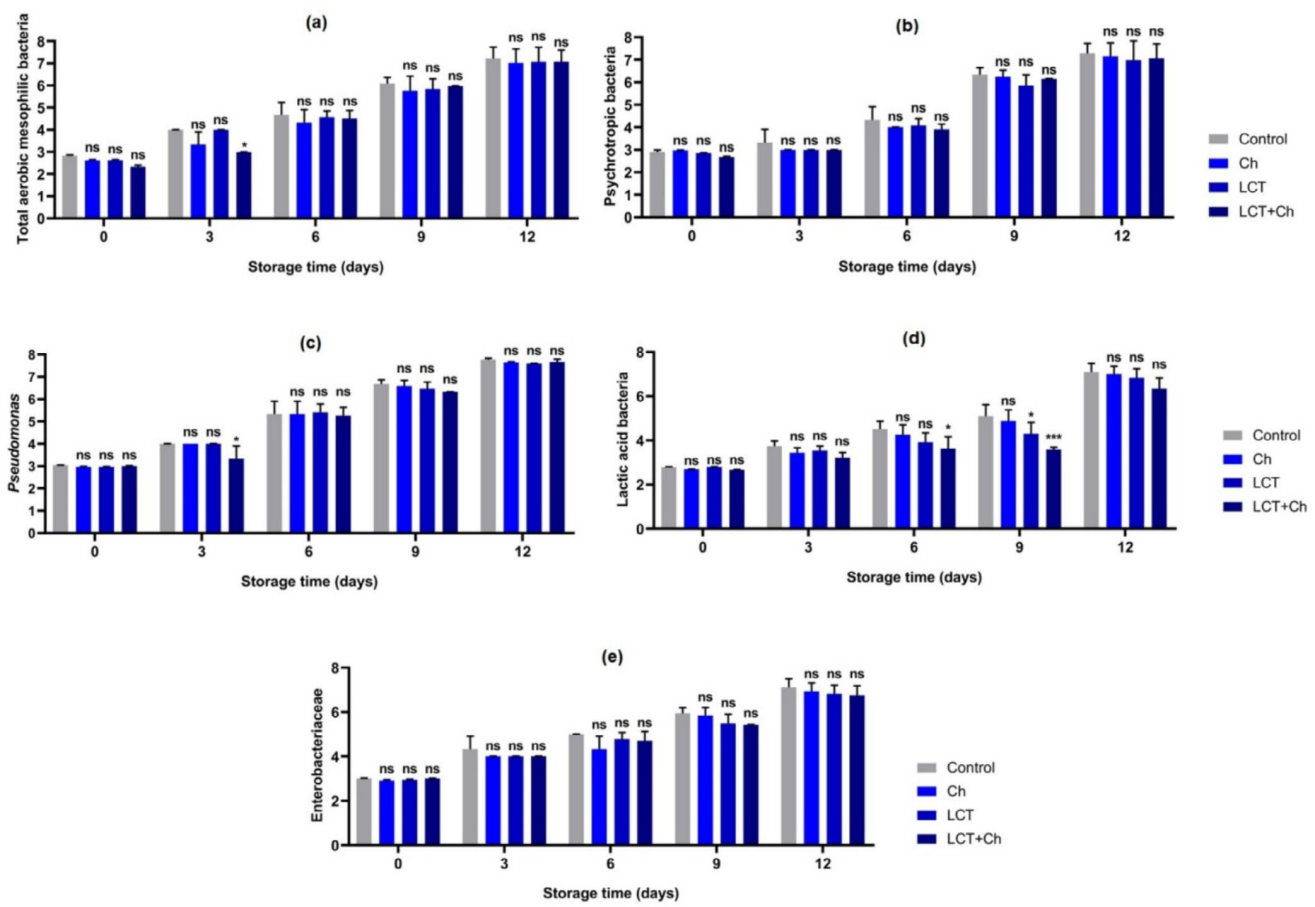
Effect of fish mucus LCT treatments on microbiological status (log CFU/g) of rainbow trout fillets during the storage time. Results were expressed as mean ± SD. ***p < 0.001, *p < 0.05, and ns: non‐significant (*p* > 0.05). Ch: chitosan; LCT + Ch: lectin + chitosan; LCT: lectin.

Due to the consumer awareness that has developed in recent years, the interest in food coatings in which different herbal and organic origin natural substances are used due to their antioxidant/antimicrobial content has increased, and much research has been carried out (Alak et al. 2023, 2019; Güler et al. 2022). However, no studies have investigated the potential food preservative effects of LCT obtained from animal origin. The present study used LCT as a food preservative in biofilm coatings for the first time based on its animal origin and antimicrobial potential. Considering the findings obtained, it is determined that the single and integrated use of LCT with chitosan is significantly (*p* < 0.05) effective on microbiological parameters on shelf life (Figure 3).

The results of total aerobic mesophilic bacteria, psychrotrophic bacteria, lactic acid bacteria, Enterobacteriaceae, and *Pseudomonas* bacteria determined during cold storage (4°C ± 1°C) in rainbow trout fillets treated with LCT enriched coating solutions are given in Figure 3. As shown in Figure 3, an increase in the number of target bacteria in all groups is determined as the storage period proceeds. In terms of total aerobic mesophilic bacteria count (Figure 3a), the highest increase in the treatment groups is observed in the control group. On the 9th day of storage, the number of bacteria in the control group reached 6–7 log CFU/g, which is accepted as the limit value. A similar trend is determined in the number of psychrotrophic bacteria (Figure 3b), and again, an increase is recorded in parallel with the increase in storage time.

**FIGURE 4:**
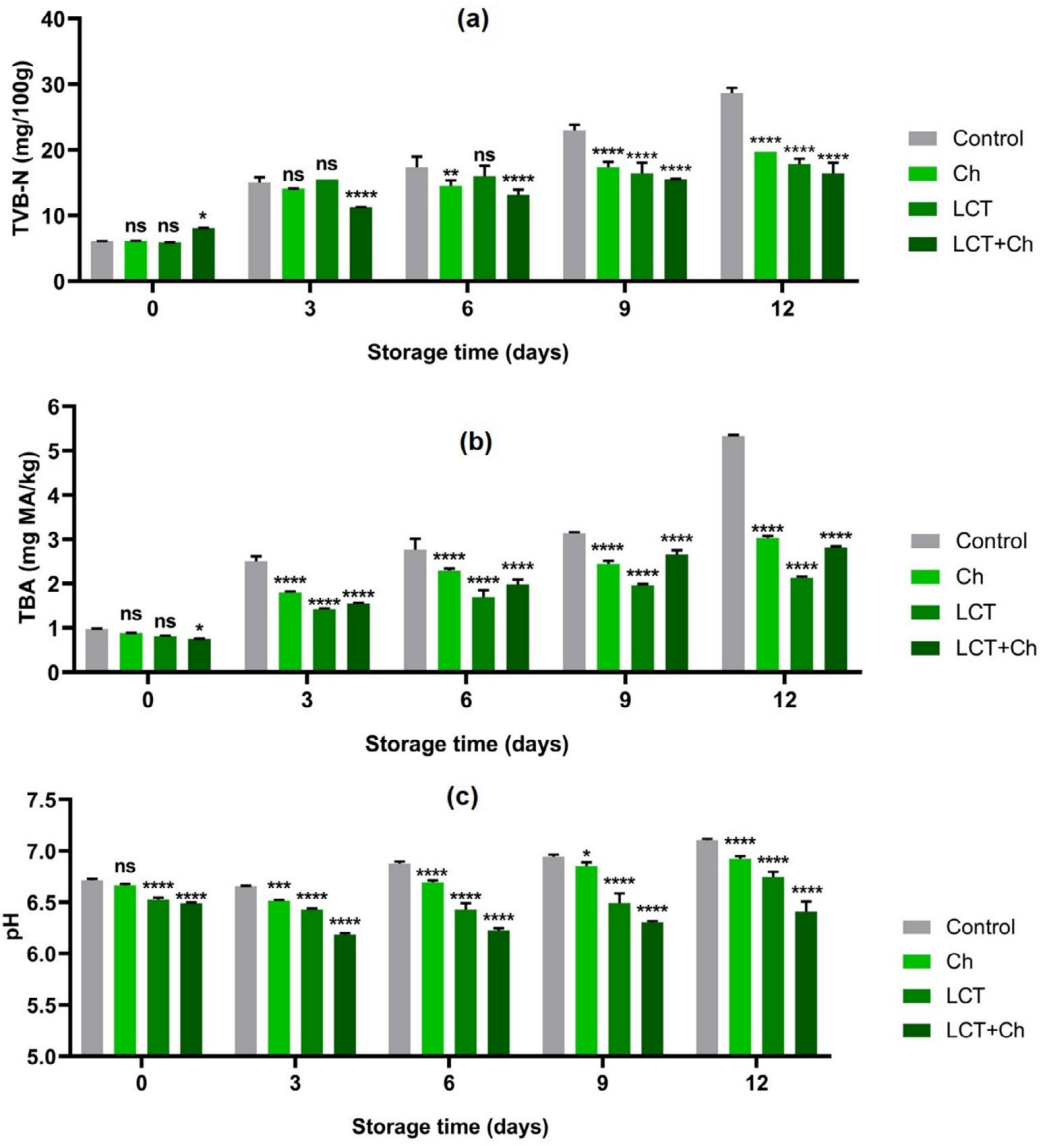
Effect of fish mucus LCT treatments on chemical parameters level of rainbow trout fillets during the storage time. Results were expressed as mean ± SD. ***p < 0.001, *p < 0.05, and ns: non‐significant (*p* > 0.05). Ch: chitosan; LCT + Ch: lectin+ chitosan; LCT: lectin.

While the number of lactic acid bacteria (LAB) (Figure 3d) is 10^2^ CFU/g in all groups at the beginning of storage (day 0), the highest number of LAB is detected at 10^7^–10^6^ CFU/g on the last day of storage as Control > LCT = Ch > LCT + Ch, respectively, and the number of LAB increased as the storage period progressed. When Enterobacteriaceae (Figure 3e) and *Pseudomonas* counts (Figure 3c) are analyzed, the lowest levels in both bacterial groups are detected in the LCT and LCT + Ch groups. When the chemical parameters are taken into consideration, the pH, TVB‐N, and TBA values of the groups in which LCT is applied alone or in combination with Ch (LCT + Ch) showed significant decreases at the end of storage compared to the group with chitosan alone (Ch) (Figure 4). Figure 4a shows the increase in TVB‐N value during storage; it appeared significantly higher in the control group than the others from Day 3 to Day 12 (*p* < 0.05). In Figure 4b, TBA values of all samples exhibited various rates of increase during storage; however, the most dramatic increases are found in the uncoated control group (*p* < 0.05). Although the initial TBA value in this group is 0.97 mg MDA/kg, this value peaked at 5.34 mg MDA/kg at the end of storage, and the other groups are determined as 3.02 mg MDA/kg (Ch), 2.13 mg MDA/kg (LCT) and 2.82 mg MDA/kg (LCT + Ch).

Figure 4c shows how the pH of the fillets has changed during the storage. The pH values, which are in the range of 6.7–6.5 at the beginning of storage, are found to be highest (7.10) in the control group and detected lowest (6.40) in the LCT + Ch group at the end of 12 days of storage (*p* < 0.05).

**FIGURE 5:**
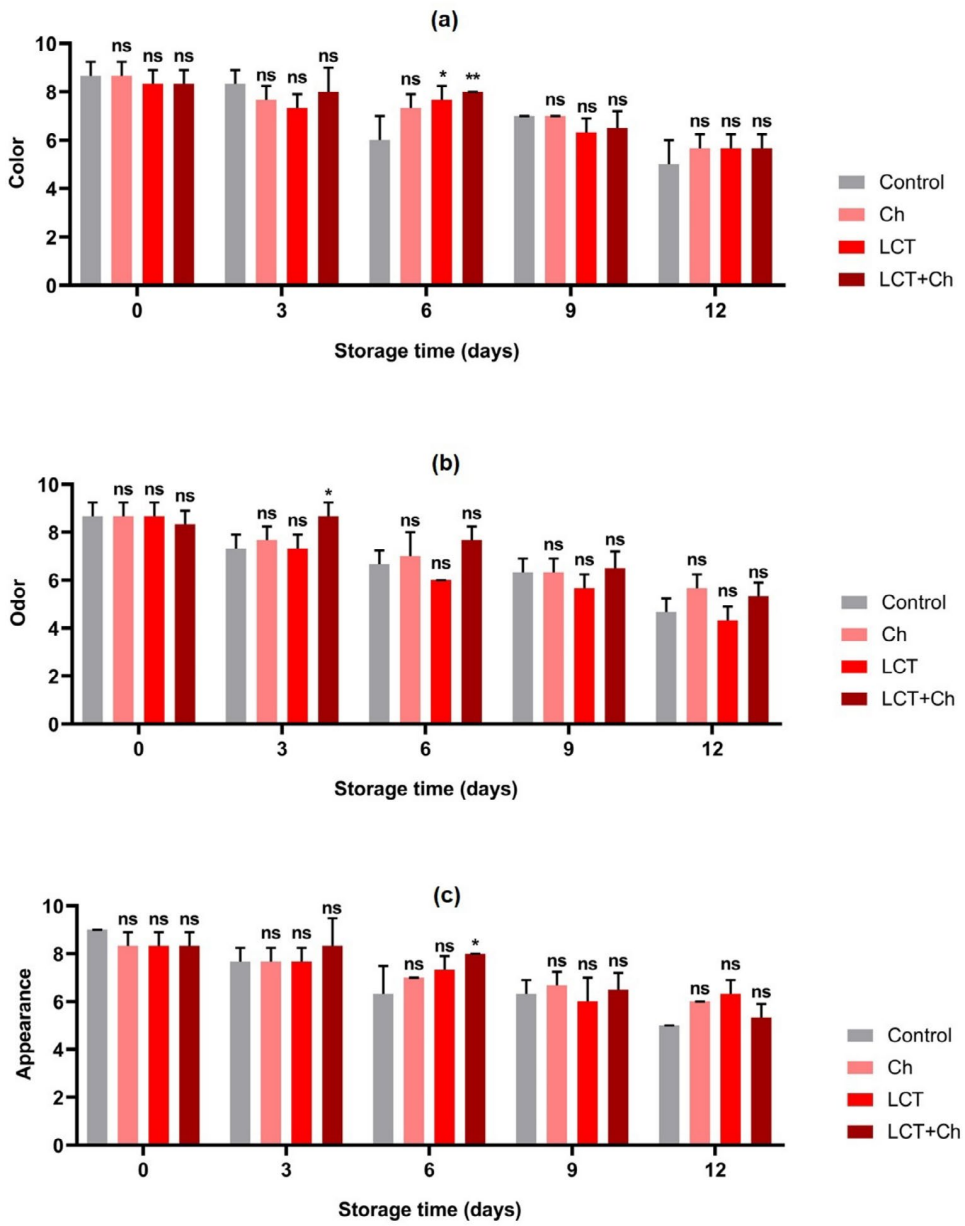
Effect of fish mucus LCT treatments on sensory score of rainbow trout fillets during the storage time: (a) Color, (b) odor, and (c) appearance. Results were expressed as mean ± SD. *p < 0.05 and ns: non‐significant (*p* > 0.05). *Mean ± standard error *n* = 7, 1–3 (very poor—unacceptable), 4–5 (fair), 6–7 (good), 8–9 (very good). Ch: chitosan; LCT + Ch: lectin + chitosan; LCT: lectin.

Sensory analyses are determined by hedonic scale, and differences in color, odor, and appearance are recorded in parallel with storage time; the appreciation in these parameters decreased gradually with time (Figure 5).

As shown in Figure 5, sensory scores of all groups decreased continuously as the storage time got prolonged (*p* < 0.05). Especially on the 6th day, the sensory (color, odor and general appearance) score of the control samples decreased, indicating that deterioration started in this group of fillets. Regarding hedonic scores for color, odor, and overall acceptability, all treatment group fillets exhibited good to very good acceptability up to Day 6. After 6 days of storage, controls began to show the symptoms of deterioration represented. Considering Figure 5, it has been determined that LCT has a significant effect, particularly on odor.

**FIGURE 6:**
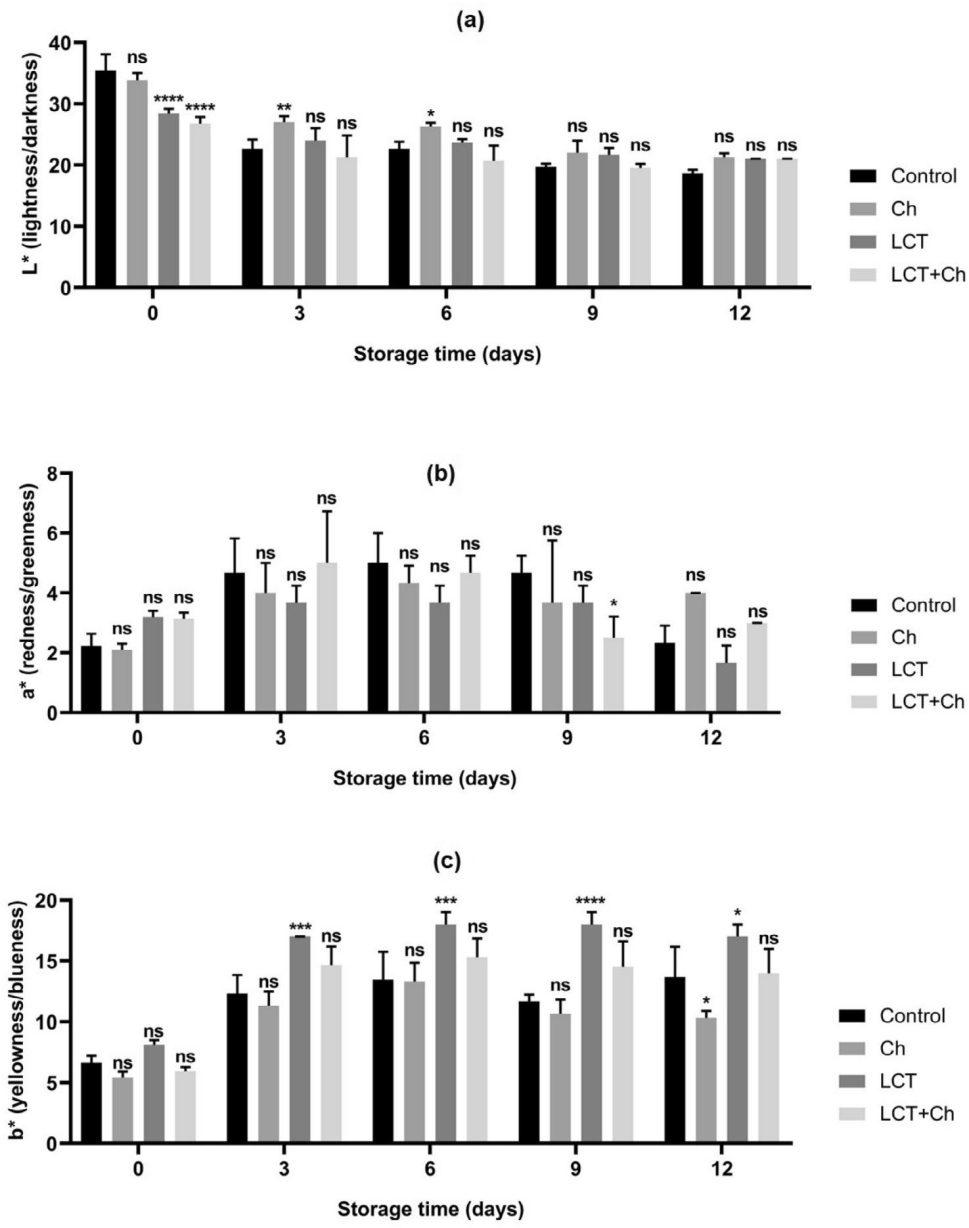
Effect of fish mucus LCT treatments on color of rainbow trout fillets during the storage time: (a) L* (lightness/darkness), (b) a* (redness/greenness), and (c) b* (yellowness/blueness). Results were expressed as mean ± SD. ****p < 0.0001, ***p < 0.001, **p < 0.01, *p < 0.05, and ns: non‐significant (*p* > 0.05). Ch: chitosan; LCT + Ch: lectin + chitosan; LCT: lectin.

The results of the color (L*, a*, and b*) evaluation of the treatment groups are presented in Figure 6. In terms of L* (lightness/darkness), a* (redness/greenness), and b* (yellowness/blueness) color values of fillets coated with LCT and LCT + CH, changes in L*, a*, and b* values are determined in trout fillets coated with LCT and/or Ch during the refrigerated storage period; however, no significant difference is detected in a* values (*p* > 0.05).

## 4 Discussion

*Lectins are generally purified* by precipitation with organic solvents or salts and using different chromatographic techniques. However, these methods present difficulties in large‐scale applications (Kara et al. 2024). Considering that the purification strategy determines 50%–90% of the production costs of biological products, there is a need for efficient, effective, and economical bioseparation techniques that can provide high purity and high recovery while preserving the biological activity of the molecule (Alves et al. 2000; Rosa et al. 2007). Alternative purification techniques developed to meet these criteria include membrane technology, affinity precipitation, magnetic separation, and partitioning into two or more immiscible aqueous phases (Nascimento et al. 2013; Yan et al. 2018). These methods also enable the purification of target products directly from crude process broths or extracts in a single process by combining the three classical steps of clarification, concentration, and initial purification (Kara et al. 2025). TPP, one of these alternative methods, has successfully purified many macromolecules such as proteins and enzymes (Kansal et al. 2006). There have been studies on the purification of lectins with TPP and aqueous two‐phase systems (Alves et al. 2000; Soares et al. 2011; Nascimento et al. 2010; Habib et al. 2024). In this study, lectin from fish mucosa was purified 7.3‐fold with 400% yield by the TPP method under optimized conditions.

Based on the molecular weights determined for mucus, it has been concluded that high MAs increase the rate of parasite infestation and mortality in fish. In contrast, low molecular weights are closer to lysozyme and have more antimicrobial properties. Along with contributing to the innate immune system of an organism, these lysozymes are more effective against gram‐positive bacteria due to the presence of peptidoglycans in their cell walls (Hussain and Sachan 2023). Our present study determined a low molecular weight of approximately 73 kDa. Habib et al. (2024) attributed the 50 kDa protein detected in one study to the presence of carbonic anhydrase, an important component of the innate immune system, which is lower than the present study value. In a different study, proteins from 3 fish species (trench, eel, and rainbow trout) were purified, and their masses as novel antibacterial proteins were recorded as 49, 45, and 65 kDa, respectively (Kilercioğlu 2021). The differences in protein concentration recorded in this and similar studies may be due to their mucus collection techniques, sex, age, habitat, water quality parameters, species, stress, and seasonal variation (Akan 2011).

LCT and LCT + Ch applications are found to be more effective in microbial inhibition as determined by our research findings. The probable reason for this situation is that the lectins, which are extracellular and soluble, recognize the molecular patterns in the specialized carbohydrate structure on the surface of the pathogenic microorganism and form a bond. Due to the established lectin bond, the microorganisms can be interpreted as macrophages and complement‐mediated cell lysis and phagocytosis (Akan 2011). Antimicrobial peptides (AMPs), which are produced by many organisms for defense against pathogens, are important components of the immune system and show strong antimicrobial effects on microorganisms. The outer surface of the bacterial cell membrane is covered with phospholipids with a negative charge. AMPs attach to this phospholipid surface thanks to their positive charge and vertically transfer their hydrophobic residues into the membrane. After transfer, both the structural integrity of the membrane is disrupted and the selective permeability of the membrane is lost. Thus, bacterial growth is inhibited by disrupting the balance between the cell's internal and external environment (Akar and Uyanıkgil 2020; Ma et al. 2024). Similarly, lectins can bind to the cell surface and disrupt the membrane integrity of microorganisms. In particular, some C‐type lectins are known to cause membrane destabilization by forming pores on the membrane similar to AMPs (Mukherjee et al. 2014). In a study by Lordache et al. (2015), it was reported that lectins showed antibacterial and antifungal activity against Gram‐negative and Gram‐positive bacteria and fungi by binding to peptidoglycans, polysaccharides, lipopolysaccharides (LPSs), teichoic, and teichuronic acids found in bacterial and fungal cell walls. Furthermore, another study by Fernández‐Alonso et al. (2012) indicated that lectins can bind to carbohydrates by strong forces such as hydrogen bonds, hydrophobic, and electrostatic interactions. It has been reported that LCT mediates killing by binding to carbohydrate structures on the surface of many bacterial, fungal, and viral cells, activating the complement system or acting like opsonin and increasing phagocytosis; and it is noted that the protein in question is activated as a result of a series of biological reactions and leads to the lysis of the microorganism (Yıldırım et al. 2015). In one study, it has been reported that crude and aqueous mucus showed bactericidal activity against *E. coli* , *A. thallasinus*, *P. aeruginosa* , and *A. maculatus* (Hussain and Sachan 2023). Likewise, the possible mechanism for the strengthening of this inhibition in LCT + Ch groups can be attributed to the presence of free amino groups in the chitosan structure. If the pH value of the medium is lower than the *p*K_a_ value (6.3–6.5) of the amino groups of chitosan and its derivatives, chitosan and its derivatives acquire a polycationic structure (Alak et al. 2010). It has been reported that the main role in antibacterial activity is performed by the electrostatic interaction between chitosan in polycationic form and anionic components (e.g., lipopolysaccharides and cell surface proteins in Gram‐negative bacteria) on the surface of microorganisms. As a result of electrostatic interaction, the distribution of negative and positive charges on the cell surface is differentiated, and thus, membrane stability deteriorates and permeability changes. With the change in membrane permeability, nutrients cannot enter the cell, or intracellular components leak out of the cell and cell death occurs as a result. When the pH value of the medium is higher than the *p*K_a_ value of chitosan, the mechanism responsible for antibacterial activity is due to hydrophobic interaction and chelating effect rather than electrostatic interaction. Owing to the difference in the cell walls of Gram‐negative and Gram‐positive bacteria, the antibacterial mechanism of action of chitosan may be different in such bacteria (Guo et al. 2008). Considering the molecular weight of the chitosan obtained in our study, the observed inhibition mechanism is believed to differ based on the chitosan's molecular weight (Huang et al. 2024). According to Guo et al. (2008), high molecular weight chitosan is likely inhibited by adsorbing onto the cell wall, whereas low molecular weight chitosan penetrates the cell and exerts its inhibitory effect internally. Studies have shown that Ch controls the growth of microorganisms in seafood, delaying quality deterioration and extending shelf life to a certain extent (Izadi et al. 2024).

In this study, the carbohydrate‐based structure of chitosan (N‐acetyl‐D‐glucosamine), which exhibits rich bioactive properties with its biopolymer properties and cationic structure, is thought to significantly increase and support the antimicrobial activities of lectins by binding to carbohydrate molecules.

Chitosan not only has a structure that enhances biological activity, but also stands out as a functional component that reinforces the interaction of lectins on the membrane of microorganisms. In this context, the antimicrobial activity of chitosan creates a synergistic effect that optimizes the interaction of lectins with microorganisms (Kou et al. 2022; Aranaz et al. 2021; Ke et al. 2021).

In the research findings, the pH increase observed for all groups may be due to the accumulation of alkaline compounds such as ammonia produced by the action of microorganisms and endogens. On the other hand, the low pH values determined in LCT + Ch treatment in our study can be explained by the inhibition of endogenase protease activity. Such a situation conforms to the results of our microbiological analyses. During the storage period, a considerable increase in the pH of all samples is probably due to bacterial growth causing the degradation of amino acids and the production of amino group degradation by‐products (such as ammonia, aldehydes, and ketones) during storage and enzymatic degradation of cellular contents (Abdel‐Naeem and Mohamed 2016). The slight pH increases determined in the LCT single and integrated (with Ch) treatment groups compared to the other treatments are attributed to the inhibitory effect of the film coating against gas permeability as well as the decrease in enzyme activity and microbial growth attributed to the antimicrobial properties of LCT and/or Ch (Yıldırım et al. 2015).

The accumulation of volatile substances such as ammonia, trimethylamine, and alkalis resulting from the activity of microorganisms and endogenous enzymes during storage caused an increase in pH (Izadi et al. 2024). Nevertheless, the results revealed that the biological coating can delay the growth of basic and acidic molecules by showing an effective barrier property and that the antibacterial activity of Ch and LCT has an effect on these coating groups.

TVB‐N is an important indicator for assessing the freshness of fish fillets and is an important parameter related to the degree of degradation of proteins and nucleic acids as a consequence of microbial spoilage (Yang et al. 2024). The increase in TVB‐N determined in all treatment groups during storage means the accumulation of basic nitrogen‐containing substances such as protein decomposition, ammonia, dimethylamine, TMA, and some other volatile basic nitrogen compounds, among which TMA is produced by microbial enzymes that degrade trimethylamine oxide (TMAO) (Izadi et al. 2024). The striking increase observed during the storage period, especially in the control group, may be associated with spoilage‐causing bacteria switching from glycogen‐dependent bacteria to protein‐degrading bacteria (Kou et al. 2022). On the other hand, low TVB‐N values determined in LCT + Ch, Ch, and LCT groups indicate a slower degradation rate of bacteria, which are effective in protein degradation in these treatments. Additionally, the antibacterial activity observed by the synergistic effect of LCT + Ch resulted in a lower TVB‐N value, which can be attributed to the inhibition of protein decomposition supported by the total number of aerobic mesophilic bacteria.

TBA is an indicator that reflects the freshness of the fish and can reflect the level of lipid oxidation in the tissues (Izadi et al. 2024; Abdel‐Naeem and Mohamed 2016). At the end of the storage, very low values are obtained in the coated groups compared to the control. The findings suggest that the film coating caused a significant decrease in the rate of increase of TBA and that the coating slowed the oxidative rate by reducing oxygen diffusion and inhibiting lipid oxidation (Izadi et al. 2024). Besides, the increased TBA levels during storage may have occurred depending on the loss of moisture and oxidation of unsaturated fatty acids during storage (Abdel‐Naeem and Mohamed 2016).

High levels of TVB‐N lead to changes in texture and the appearance of unpleasant odors and tastes (Huang et al. 2024; Sayyari et al. 2021; Zhang et al. 2024). With a gradual decrease in the sensory score depending on the storage time, it has been determined that the fillets have organoleptic acceptance values at a medium level. The highest rate of decrease in the scores has been observed in the control group, which supports this deterioration in the TVB‐N and TBA values of this group. It is well known that an increase in TBA is accompanied by a decrease in the quality of fish fillets and changes in taste and color. A decrease in the sensory score is usually correlated with the proliferation of microorganisms, protease enzymolysis, and lipid oxidation in the tissue (Izadi et al. 2024). Similarly, pH increases have adverse effects on the color, taste, texture, and shelf‐life quality of fish during frozen storage (Abdel‐Naeem and Mohamed 2016). When the chemical and microbiological data are combined with the sensory scores, a putrid fishy taste appears in the control group on day 12; this is probably due to the TVB‐N approaching the acceptable limit (Izadi et al. 2024).

Color is the most important quality attribute directly associated with freshness, and therefore the most important quality attribute in consumer perception, and as the freshness of foods decreases, discoloration increases (Bonilla et al. 2018). Various factors, such as the physical structure of fish fillets, pigment content, and free water content, significantly affect the color properties. Enzymatic activity during frozen storage changes the structure of myofibrillar proteins, and protein oxidation affects light scattering and causes changes in L* values (Alak et al. 2023). With prolonged storage time, fillet color may decrease to different degrees due to microbial activity causing protein degradation during storage. Under the influence of oxygen, myoglobin oxidizes to bronze‐colored high‐iron myoglobin and bright red oxygenated myoglobin (Zhang et al. 2024). The yellow discoloration of fillets during refrigerated storage may not be related to carotenoid content, because the chemical change that causes darker and more yellow pigmentation in this type of storage is still unknown. Also, the overall effect on redness (a*) during storage can be explained by the inherent low pigmentation of the fish (Bonilla et al. 2018).

The total dietary intake of lectins varies between approximately 0 and 200 mg per day (Lucius 2020). Regarding the concerns about lectin intake in diets, the small amount used in the study and the storage of seafood with different processing technologies compared to vegetables and the use of different cooking techniques may reduce these concerns and have a positive effect on human health. In general, lectins, which are stable against heat denaturation and proteolytic digestion, show different reactions to degradation by intestinal enzymes. It has been reported that vegetables and fruits, in particular, contain significant amounts of lectins and are consumed in an inactive form and are still safe to eat. It has been reported that lectins may have beneficial effects even with low dietary intake and do not have a measurable negative effect on nutritional activity (Buendia et al. 2024). However, it has been reported that many proteins, including lectins, exhibit anticancer, anti‐inflammatory, and immunostimulatory properties together with peptides such as lunasin and regulate microbiota flora and metabolite production (Benavides‐Carrasco and Jarpa‐Parra 2024).

Considering this situation, more studies on fish mucus LCT are needed to generalize and draw accurate conclusions due to the consideration of shelf life in fish. In these studies, the suggestion that especially low concentrations (< 0.04 mg) should be tested can be presented as our study data. However, in order to demonstrate its effectiveness, it should be studied in different foods (processed and unprocessed) with different concentrations and integrations.

## 5 Conclusion

There has been increasing interest in the utilizing of food wastes and by‐products and their industrial use as antioxidants and antimicrobials to extend the shelf life of foods. Based on literature research, the application of a potential preservative originating from aquaculture products for the protection and/or quality improvement of the final product has not been encountered, especially in aquaculture processing technologies or packaging techniques, and the current study is the first in this sense. Based on the existing literature, the findings innovatively modeled the biological activities and potential applications of LCT purified from mucus for the aquaculture processing sector. Various antimicrobial compounds in fish mucus, such as lectin, can be purified and used against microorganisms that have adapted or modified to resist antibiotics for all types of biofilm coating. Compounds extracted from mucus can also be used synergistically with different polymer carriers. Since LCT and different compounds extracted from mucus are natural products exhibiting multiple biological activities in functional food industries and have significant potential in various fields, they can be used as food preservatives in the food industry, especially in the aquaculture processing sector. Overall, the findings provide a solid basis for a more rapid and holistic approach to integrating LCT into edible coatings as an effective food preservative to prevent spoilage and increase shelf life and fillet quality. Thus, in the food field, this promising approach offers that LCT can be applied as an additive and find a good motivation potential in the interaction between producers and consumers in developing relevant products.

Limitations and future perspectives of the study:

- Obtaining suitable and sufficient waste/by‐product biomass.
- Extraction methodology.
- Successfully determining the physical and chemical properties of synthesized products and their integrations with the food industry through rigorous testing.

Although there are the limitations mentioned above, the expected perspective for the sector requires more effective use of resources. Within the scope of green and sustainable agricultural practices, the formation of effects aimed at utilizing seafood waste/by‐products for bio‐food preservative solutions, with clear usage scenarios for new and innovative‐origin product acquisition technology and providing “Social Innovation in the Environment”. Additionally, the economic recovery of rainbow trout processing waste, converting these wastes into new, functional, and environmentally friendly applications in the food industry, enabling their mass production as alternative food preservatives. This will foster motivation by encouraging new studies, promoting the economic recovery of seafood processing waste with a value‐added product profile, and examining the effects of products developed from these wastes in the experimental production model across various food industry sectors.

## Author Contributions

**Melda Şişecioğlu:** formal analysis (equal), investigation (equal), methodology (equal), resources (equal), supervision (equal), writing – original draft (equal). **Rahime Altıntaş:** formal analysis (equal), investigation (equal), methodology (equal), resources (equal), validation (equal). **Ayşe Kara:** formal analysis (equal), investigation (equal), methodology (equal), resources (equal), validation (equal). **Gonca Alak:** conceptualization (equal), data curation (equal), formal analysis (equal), investigation (equal), methodology (equal), project administration (equal), resources (equal), supervision (equal), writing – original draft (equal), writing – review and editing (equal).

## Conflicts of Interest

The authors declare no conflicts of interest.

## Data Availability

The data that support the findings of this study are available from the corresponding author upon reasonable request.
