# Correction to “Utilization of Fish Byproduct in Edible Films: A Sustainable Approach to Improving Fillet Shelf Life”

**DOI:** 10.1002/fsn3.70813

**Published:** 2025-08-18

Alak, G., Kara, A., Altıntaş, R. and Şişecioğlu, M. (2025). Utilization of Fish Byproduct in Edible Films: A Sustainable Approach to Improving Fillet Shelf Life. *Food Science & Nutrition*, 13: e70731. https://doi.org/10.1002/fsn3.70731

The following corrections are noted:

In Table 1, “A: Ammonium sulfate” should be changed to “Ammonium sulfate concentration” and “B: t‐butanol” should be changed to “Supernatant/t‐butanol ratio.” and “C: pH” should be changed “pH”. “C” remove same “A” and “B”.

In section 2.2, the equation:

$$y=\left(454.8+66.6A-26.5B+49.0C-139.9A*A+89.7B*B-96.8C*C+94.7A*B-35.9A*C-11.5B*C\right)$$

should be changed to:

$$y\left(\%\right)=\left(454.8+66.6A-26.5B+49.0C-139.9A*A+89.7B*B-96.8C*C+94.7A*B-35.9A*C-11.5B*C\right)$$

In section 2.3, the equation:

$$Y=\frac{{\mathrm{HA}}_{I\;}.V_{I}}{{\mathrm{HA}}_{H\;}.V_{i}}\times 100$$

should be changed to:

$$Y\left(\%\right)=\frac{{\mathrm{HA}}_{I\;}.V_{I}}{{\mathrm{HA}}_{H\;}.V_{i}}\times 100$$

In section 2.4, “Polyacrylamide Gel Electrophoresis” should be changed to “Sodium Dodecyl Sulfate‐Polyacrylamide Gel Electrophoresis.”

In section 3.1, the following sentence should be added to the end of the paragraph: “The best hemagglutination activity of the mucus extraction was determined in the extract with a concentration of 1/64.”

We apologize for this error.

